# Nfatc1 Is a Functional Transcriptional Factor Mediating Nell-1-Induced Runx3 Upregulation in Chondrocytes

**DOI:** 10.3390/ijms19010168

**Published:** 2018-01-06

**Authors:** Chenshuang Li, Zhong Zheng, Xinli Zhang, Greg Asatrian, Eric Chen, Richard Song, Cymbeline Culiat, Kang Ting, Chia Soo

**Affiliations:** 1 Division of Growth and Development, Section of Orthodontics, School of Dentistry, University of California, Los Angeles, Los Angeles, CA 90095, USA; chenshuanglee@gmail.com (C.L.); zzheng@dentistry.ucla.edu (Z.Z.); xinli@ucla.edu (X.Z.); gasatrian@ucla.edu (G.A.); ericchenucla@g.ucla.edu (E.C.); richardsong227@gmail.com (R.S.); 2 NellOne Therapeutics, Inc., 99 Midway Ln # E, Oak Ridge, TN 37830, USA; cymbeline@nell-one.com; 3 Division of Plastic and Reconstructive Surgery and Department of Orthopaedic Surgery, the Orthopaedic Hospital Research Center, University of California, Los Angeles, Los Angeles, CA 90095, USA

**Keywords:** neural EGFL like 1 (Nell-1), nuclear factor of activated T-cells 1 (Nfatc1), runt-related transcription factor 3 (Runx3), chondrogenesis

## Abstract

Neural EGFL like 1 (Nell-1) is essential for chondrogenic differentiation, maturation, and regeneration. Our previous studies have demonstrated that Nell-1’s pro-chondrogenic activities are predominantly reliant upon runt-related transcription factor 3 (Runx3)-mediated Indian hedgehog (Ihh) signaling. Here, we identify the nuclear factor of activated T-cells 1 (Nfatc1) as the key transcriptional factor mediating the Nell-1 → Runx3 signal transduction in chondrocytes. Using chromatin immunoprecipitation assay, we were able to determine that Nfatc1 binds to the −833–−810 region of the *Runx3*-promoter in response to Nell-1 treatment. By revealing the Nell-1 → Nfatc1 → Runx3 → Ihh cascade, we demonstrate the involvement of Nfatc1, a nuclear factor of activated T-cells, in chondrogenesis, while providing innovative insights into developing a novel therapeutic strategy for cartilage regeneration and other chondrogenesis-related conditions.

## 1. Introduction

A diverse group of molecules is involved in the regulation of chondrogenic differentiation, an essential process for bone and cartilage formation [1]. Previous studies demonstrated that neural EGFL like 1 (Nell-1) enhanced chondrogenic marker expression and cartilage nodule formation in rabbit chondrocytes [2]. Additionally, Nell-1 administration has been observed to induced hyaline cartilage regeneration in a rabbit knee subchondral defect model [3], and implantation of Nell-1-overexpressing bone marrow mesenchymal stem cells into critical-sized goat mandibular condyle osteochondral defects resulted in articular cartilage reestablishment [4]. Conversely, homozygous *N*-ethyl-*N*-nitrosourea (ENU)-induced Nell-1-deficient mice fail to survive perinatally, accompanied by shorter and deformed rib cages and vertebral bodies, compressed intervertebral spaces, and reduced expression of cartilage matrix in comparison with those of the wild-type and heterozygous littermates [5]. Our recent studies demonstrated that, in chondrocytes, the expression of Nell-1 is regulated by the runt-related transcription factor 2 (Runx2) [6], a pivotal transcriptional factor regulating chondrogenesis [7]. Interestingly, Nell-1’s pro-chondrogenic activities extended to *Runx2^−/−^* scenarios by stimulating the expression and signal transduction of runt-related transcription factor 3 (Runx3) and its downstream target Indian hedgehog (Ihh) [6,8]. 

Previous findings suggested that *Runx3* is not one of the early response genes during Nell-1-regulated chondrogenesis [9]. As such, it is critical to identify the molecule(s) mediating the Nell-1-stimulated *Runx3* expression to further elucidate the mechanism of action of Nell-1 in chondrogenesis. In this study, we first screened key transcriptional factors bridging the Nell-1 → Runx3 signal transduction using a two-step in silico promoter analysis and quantitative real-time PCR (qPCR) strategy. These findings were further validated using RNA interference (RNAi) technology. Subsequently, we used chromatin immunoprecipitation (ChIP) assay to recognize the binding site of the identified key Nell-1-induced transcriptional factor on the *Runx3* promoter. The current study is not only an additional new section of a series of investigations that uncover the underlying mechanism of Nell-1’s function in chondrogenesis, but also demonstrates the involvement of Nfatc1, a nuclear factor of activated T-cells, in chondrogenesis, while providing innovative insights that will assist in the development of a novel therapeutic strategy for cartilage regeneration and other chondrogenesis-related conditions.

## 2. Results

### 2.1. Runt-Related Transcription Factor 1 (Runx1) and Nfatc1 Were Selected as the Candidates That Bridge Nell-1 → Runx3 Signal Transduction in Chondrocytes

Using in silico bioinformatics software Genomatix to predict the binding candidates of mouse *Runx3* promoter, we identified 22 cartilage/chondrocyte-expressed transcriptional factor candidates (Table 1). Compared with previous microarray data that screened the primary response genes of Nell-1-mediated chondrogenesis (GEO DataSet: GSE23570) [9] as a reference, it was observed that *runt-related transcription factor 1* (*Runx1*), *T-cell leukemia, homeobox1* (*Tlx1*), *nuclear factor of activated T-cells 1* (*Nfatc1*; previously known as *nuclear factor of activated T-cells, cytoplasmic calcineurin-dependent 1* and also known as *Nfat2* and *Nfatc*), and *nuclear factor of activated T-cells 5* (*Nfat5*) were significantly upregulated by Nell-1 in chondrogenic-committed ATDC5 cells and were therefore selected for further investigation in the current study.

**Figure 1:**
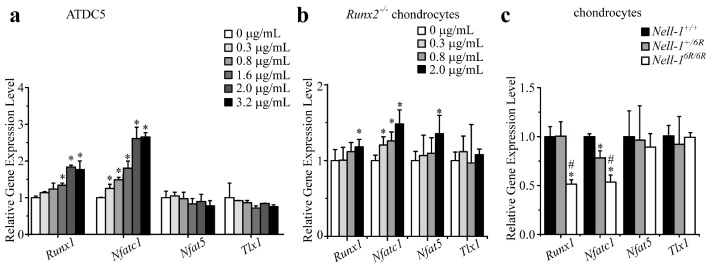
Nell-1 selectively alters the expression levels of *Runx3*-promoter binding candidates in chondrocytes. (**a**) Expression levels of *Runx3*-promoter binding candidates in monolayer cultured ATDC5 cells after stimulation with exogenous Nell-1 for 3 h. *: *p* < 0.05 vs. 0 μg/mL Nell-1; (**b**) Expression levels of *Runx3*-promoter binding candidates in monolayer cultured *Runx2^−/−^* rib chondrocytes after stimulation with exogenous Nell-1 for 12 h. *: *p* <0.05 vs. 0 μg/mL Nell-1; (**c**) Expression levels of *Runx3*-promoter binding candidates in rib chondrocytes with different genotypes of Nell-1. *: *p* < 0.05 vs. *Nell-1^+/+^* chondrocytes; ^#^: *p* < 0.05 vs. *Nell-1^+/6R^* chondrocytes. Mean + SD of three (**a**,**b**) or six (**c**) independent experiments performed in triplicate are shown.

**Figure 2:**
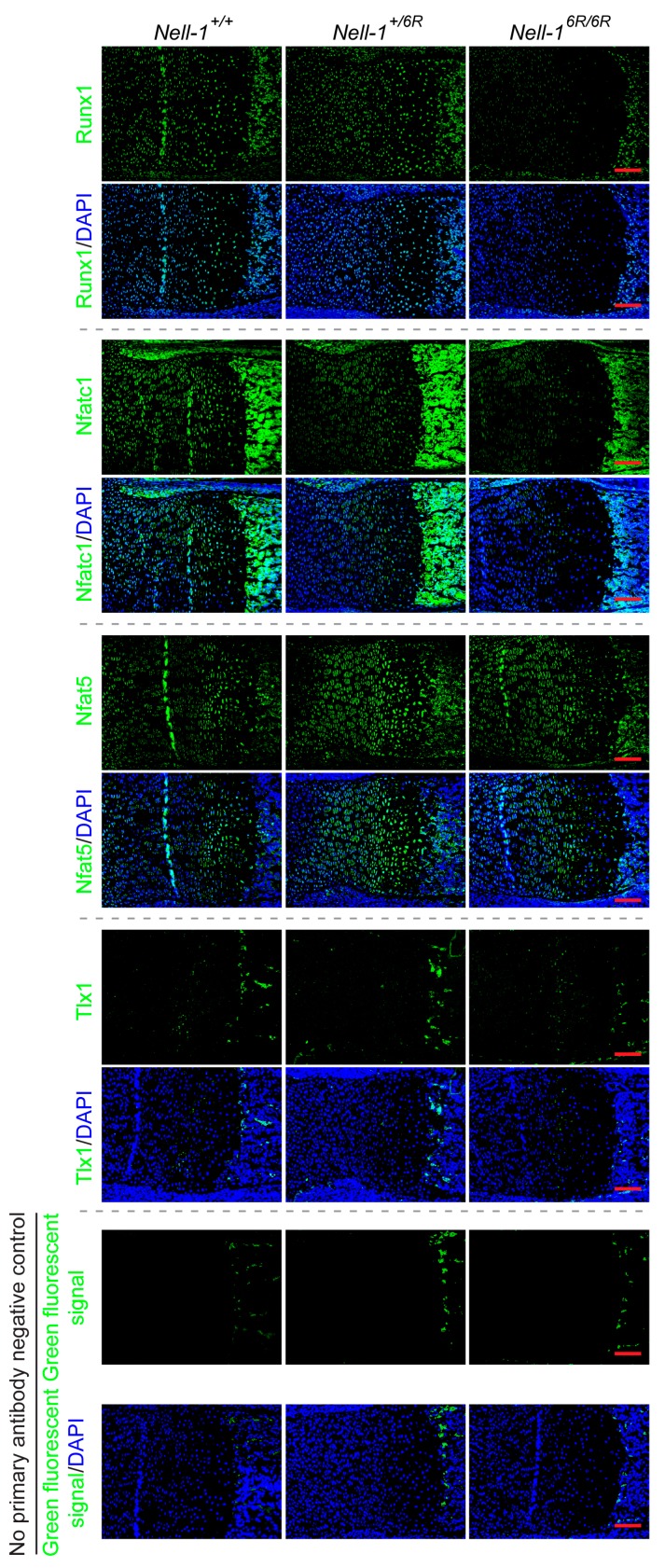
Immunofluorescent (IF) staining of *Runx3*-promoter binding transcriptional factor candidates in the femur of neonatal mice with different genotypes of Nell-1. Green, targeted molecule; blue, DAPI counterstaining. In the no primary antibody negative control, the green fluorescent signal was only detected on the red blood cells. Scale bar = 50 μm.

Although preliminary microarray assay revealed that Nell-1 upregulated the expression of all four potential candidate genes [9], qPCR demonstrated that Nell-1 only significantly elevated *Runx1* and *Nfatc1* expression in ATDC5 cells (Figure 1a). To eliminate the influence of Runx2, which could significantly induce Nell-1 expression in chondrocytes [6] and stimulate their proliferation, differentiation, and hypertrophy [10,11,12], these four Nell-1-responsive transcriptional factor candidates were further validated in *Runx2^−/−^* chondrocytes [6,8]. Like in ATDC5 cells, Nell-1 did not alter *Tlx1* expression in *Runx2^−/−^* chondrocytes (Figure 1b). Interestingly, in *Runx2^−/−^* chondrocytes, while only high dose Nell-1 slightly increased *Runx1* and *Nfat5*, all tested doses of Nell-1 upregulated *Nfatc1* expression significantly (Figure 1b). Moreover, the expression of *Tlx1* and *Nfat5* remained consistent in chondrocytes with different *Nell-1* genotypes, while expression of *Runx1* and *Nfatc1* was significantly reduced in *Nell-1^6R/6R^* chondrocytes (Figure 1c). Of the four tested transcriptional factor candidates, only *Nfatc1* was downregulated in *Nell^+/6R^* chondrocytes (Figure 1c), suggesting that *Nfatc1* is more sensitive to Nell-1 levels when compared to *Runx1*. Immunofluorescent (IF) staining confirmed a similar protein expression pattern of these candidates in the neonatal mouse femurs (Figure 2). Thus, Runx1 and Nfatc1 were selected for further investigation.

### 2.2. Knockdown Runx1 Failed to Demonstrate the Effects on the Nell-1-Mediated Runx3-Ihh Signaling

**Figure 3:**
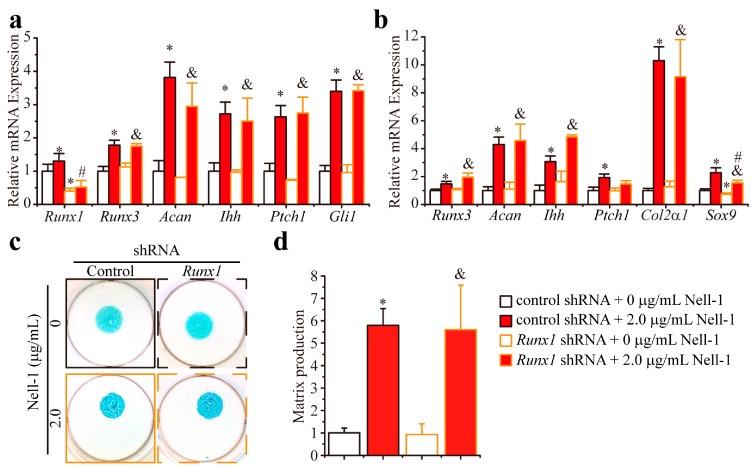
*Runx1* knockdown does not alter the Nell-1’s effects on Runx3-Ihh signaling activation and chondrogenesis of *Runx2^−/−^* rib chondrocytes. (**a**) Transcription of Runx3 and Ihh signal-related molecules in monolayer cultured *Runx2^−/−^* rib chondrocytes after 12-h stimulation of Nell-1; (**b**) Transcription of Runx3 and Ihh signal-related molecules in 3D micromass cultured *Runx2^−/−^* chondrocytes after 3-day stimulation of Nell-1; (**c**) Alcian Blue staining of 3D micromass cultured *Runx2^−/−^* chondrocytes after 3-day stimulation of Nell-1; (**d**) Alcian Blue incorporation into the extracellular matrix of micromass cultures reflecting the production of the proteoglycan-rich cartilaginous matrix at day 3 was quantified after extraction. The dye concentration of each group was normalized to that of the control shRNA + 0 µg/mL Nell-1 group. Mean + SD of three independent experiments performed in triplicate are shown. *: *p* < 0.05 vs. control shRNA + 0 µg/mL Nell-1, ^#^: *p* < 0.05 vs. control shRNA + 2.0 µg/mL Nell-1, ^&^: *p* < 0.05 vs. *Runx1* shRNA + 0 µg/mL Nell-1.

In the 2D monolayer-cultured *Runx2^−/−^* chondrocytes, *Runx1* knockdown by shRNA did not change the basal expression levels of *Runx3*, *Acan* (encoding aggrecan, a Runx3 downstream major structural component in cartilage matrix [7,13]), *Ihh, Ptch1* (encoding Ihh downstream target patched homology 1 (Patched 1)), and *Gli1* (encoding Ihh-responsive Gli1) (Figure 3a). Interestingly, Nell-1 significantly increased the expression of these genes in *Runx1*-knockdown *Runx2^−/−^* chondrocytes to levels comparable to that of the control shRNA + 2.0 µg/mL Nell-1 group (Figure 3a). Similar findings were observed in the 3D cultured *Runx2^−/−^* chondrocyte micromass: the transcription of Runx3–related (*Runx3* and *Acan*), Ihh signaling-related (*Ihh* and *Ptch1*), chondrogenic differentiation related genes *Col2α1* (encoding the α1 chain of type II collagen, an abundant and specific protein in cartilage [1,7]) and *Sox9* (encoding the master transcription factor for chondrogenesis initiation SRY-Box 9 [1,7]) (Figure 3b); also, Alcian Blue staining intensity (Figure 3c,d) was almost at the same levels as in the control shRNA + 0 µg/mL Nell-1 and *Runx1* shRNA + 0 µg/mL Nell-1 groups. Furthermore, there were no significant differences observed between the control shRNA + 2.0 µg/mL Nell-1 and *Runx1* shRNA + 2.0 µg/mL Nell-1 groups with regard to these indexes (Figure 3b–d). Thus, it can be concluded that Runx1 does not actively participate in the Nell-1 activation of Runx3-Ihh signaling in chondrocytes, at least when Runx2 is absent.

### 2.3. Nfatc1 Mediates Nell-1’s Role in Runx3-Ihh Signaling and Chondrogenic Differentiation

**Figure 4:**
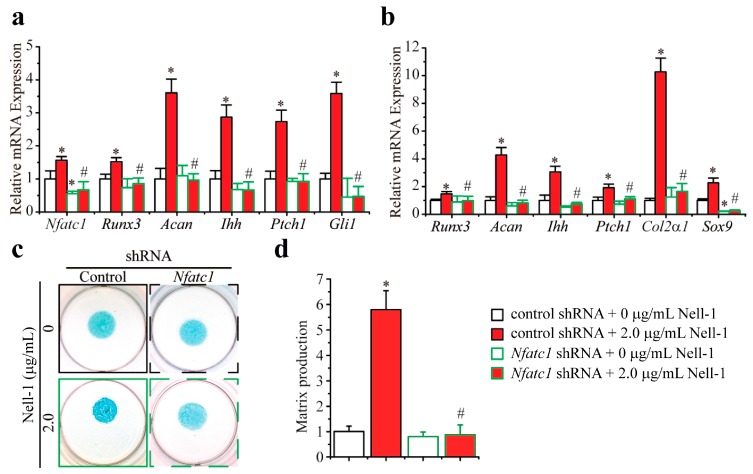
Nfatc1 is the bridging molecule between Nell-1 and the Runx3-Ihh signaling pathway in *Runx2^−/−^* rib chondrocytes. (**a**) Transcription of Runx3 and Ihh signal-related molecules in monolayer cultured *Runx2^−/−^* rib chondrocytes after 12-h stimulation of Nell-1; (**b**) Transcription of Runx3 and Ihh signal-related molecules in 3D micromass cultured *Runx2^−/−^* chondrocytes after 3-day stimulation of Nell-1; (**c**) Alcian Blue staining of 3D micromass cultured *Runx2^−/−^* chondrocytes after 3-day stimulation of Nell-1; (**d**) Alcian Blue incorporation into the extracellular matrix of micromass cultures reflecting the production of the proteoglycan-rich cartilaginous matrix at day 3 was quantified after extraction. The dye concentration of each group was normalized to that of the control shRNA + 0 µg/mL Nell-1 group. Mean + SD of three independent experiments performed in triplicate are shown. *: *p* < 0.05 vs. control shRNA + 0 µg/mL Nell-1, ^#^: *p* < 0.05 vs. control shRNA + 2.0 µg/mL Nell-1.

Although *Nfatc1* knockdown by shRNA did not significantly alter the basal expression levels of either *Runx3* and its downstream target *Acan*, or of Ihh signaling-related genes (*Ihh* and *Ptch1*) in *Runx2^−/−^* chondrocytes, Nell-1’s stimulation of these genes was completely nullified by *Nfatc1* knockdown (Figure 4a). Moreover, in the 3D cultured *Runx2^−/−^* chondrocyte micromass that underwent chondrogenic differentiation, Nell-1 was neither able to upregulate the expression of all tested genes or to enhance Alcian Blue staining in the *Nfatc1*-knockdown *Runx2^−/−^* chondrocyte micromass (Figure 4b–d). Importantly, when *Nfatc1* was knocked down by shRNA transfection in *Runx2^−/−^* chondrocytes, the expression of *Sox9* in the 3D cultured *Runx2^−/−^* chondrocyte micromass was significantly downregulated with or without the treatment of Nell-1, which indicates that the initiation of Nell-1-mediated chondrogenesis was blocked (Figure 4b). In aggregate, Nell-1’s bioactivities on Runx3-Ihh signal activation and chondrogenic stimulation were abrogated by the *Nfatc1* knockdown.

### 2.4. Nell-1 Enhances the Binding of Nfatc1 at the −833–−810 Region of Runx3 Promoter in Chondrocytes

**Figure 5:**
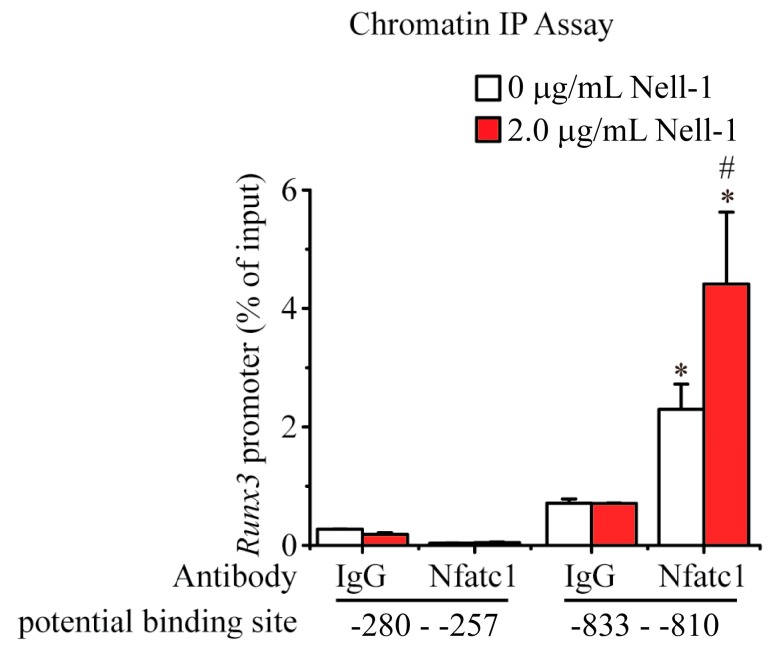
Nell-1 enhances the binding of Nfatc1 and *Runx3*-promoter in *Runx2^−/−^* rib chondrocytes. Chromatin Immunoprecipitation (ChIP) assay of Nfatc1’s binding affinity to two potential binding sites on the *Runx3* promoter was performed in *Runx2^−/^^−^* rib chondrocytes. *: *p* < 0.05 vs. IgG + 0 µg/mL Nell-1, ^#^: *p* < 0.05 vs. Nfatc1 antibody + 0 µg/mL Nell-1. Mean + SD of three independent experiments performed in triplicate are shown.

Based on the *in silico* findings, two potential binding sites of Nfatc1 were predicted on the promoter of *Runx3*: −280–−257 (ACT TTC TTT CCT TGG AGA TTT TCT) and −833–−810 (ACC TGG GTT CCA CGG TAA AGC CAG). Using both non-specific IgG and Nfatc1-specific antibody, ChIP assays were carried out. Negligible levels of DNA accumulation were observed at the −280–−257 region, regardless of treatment with Nell-1 (Figure 5). Conversely, ChIP analysis using the Nfatc1-specific antibody demonstrated enrichment of the −833–−810 fragment of the *Runx3* promoter in *Runx2^−/−^* chondrocytes, which was further enhanced by Nell-1 stimulation. Meanwhile, ChIP analysis using a negative control IgG demonstrated minimal enrichment of this DNA sequence with or without exogenous Nell-1 treatment (Figure 5). Therefore, our ChIP assays indicated that Nfatc1 binds to the −833–−810 region of the *Runx3*-promoter in chondrocytes, and this binding is significantly enhanced by Nell-1.

## 3. Discussion

Nearly two decades ago, *Nell-1* was first observed to be upregulated in prematurely fusing and fused sutural sites of craniosynostosis (CS) patients [14], suggesting Nell-1’s involvement in osteochondral development. Subsequently, our team has demonstrated that transgenic *Nell-1*-overexpression (*CMV-Nell-1*) mice recapitulate human CS-like phenotypes [15,16]. On the other hand, homozygous *Nell-1-*deficient mice were noted to exhibit neonatal lethality with reduced calvarial bone thickness and density similar to those of calvarial cleidocraniodysplastic patients [5,17,18]. These phenomena strongly indicate that Nell-1 is pivotally involved in osteogenic development.

Nell-1 also exerts pro-chondrogenic bioactivities on both mesenchymal stem cells (MSCs) and chondrogenic-committed cells [6]. Until present, however, the function of Nell-1 in chondrogenesis has not attracted enough attention, and thus the mechanism of action of Nell-1 in chondrogenic differentiation remained largely elusive. Our recent studies have shown that *Runx2* upregulates Nell-1 expression in chondrocytes, while *Runx2-*deficiency leads to significantly reduced Nell-1 levels, signifying that regulation of Runx2 has a profound impact on Nell-1 during chondrogenesis [6]. Unlike the mutual regulation between Nell-1 and Runx2 observed in osteoblast lineage cells [19,20,21], Nell-1 does not upregulate Runx2 in chondrocytes [6]. Further studies have also revealed that, as a downstream mediator of Runx2, Nell-1’s pro-chondrogenic effect relies on the activation of Ihh signaling [8]. Unlike Runx2, however, which can directly bind to *Ihh-*promoter [10,22], Nell-1-mediated upregulation of Ihh signaling was under the control of Runx3 [8], and therefore Nell-1 is able to partially rescue *Runx2*-deficiency-induced impaired chondrogenic differentiation and maturation [6,8]. 

**Figure 6:**
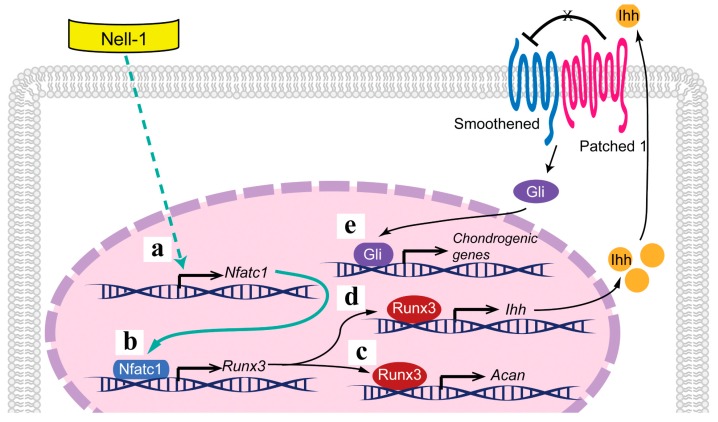
Schematic diagram of Nell-1 signaling pathways during chondrogenesis. (**a**) Nell-1 promotes Nfatc1 expression; (**b**) Nfatc1 next binds to the promoter of Runx3, activating the Runx3 signal transduction (**c**) as well as the Ihh signal transduction (**d**) to stimulate the differentiation and maturation of chondrocytes (**e**). Black arrows, previously revealed knowledge; green solid arrow, current uncovered regulation mechanism; green dashed arrow, a current suggested regulation mechanism which needs further investigation; T bar, known inhibitory effect.

In the current study, we have successfully identified and functionally validated Nfatc1 as the key transcriptional factor that bridges Nell-1 stimulation and Runx3 upregulation in chondrocytes. Nfatc1 functions as a key early Nell-1-response target in chondrocytes, upregulating *Runx3* expression via binding to its promoter at −833–−810 region and thus activating the Runx3-Ihh signal transduction cascade in order to induce chondrogenic differentiation and maturation (Figure 6). However, recent studies indicate that, as an 810-amino-acid secreted protein with multiple N-linked glycan chains, Nell-1 may function as oligomers [17]. Although the binding of Nell-1 with integrin *β*1 has been detected previously [23,24], Nell-1’s cell surface functional receptor(s) that initiates intracellular signal transduction, particularly in chondrogenic-committed cells, has yet to be discovered. Future studies through a global collaboration are warranted in order to gain a comprehensive understanding of the molecular mechanisms, especially the details of how Nell-1 stimulates Nfatc1 expression and modulates its activity. Nevertheless, the previously unrecognized Nell-1 → Nfatc1 → Runx3 → Ihh cascade provides innovative insights into developing a novel, therapeutic platform for managing cartilage regeneration and other chondrogenesis-related conditions.

Although Nfatc1–4 are all expressed in murine chondrocytes, only the regulatory effects of Nfatc1 and Nfatc2 on chondrogenesis were evaluated [25,26,27]. Since *in silico* predictions did not find the potential binding site of Nfatc2 on *Runx3* promoter, Nfatc2 was not further investigated in this study. In 2010, Sohn et al. detected the expression of Nfatc1 in the intervertebral disc of E12.5-day old mice embryos and concluded this expression was upregulated by transforming growth factor *β*1, but not bone morphogenetic protein 4, in sclerotome micromass culture [28]. In 2013, Zanotti and Canalis pioneered the investigation of Nfatc1’s function during chondrogenesis by demonstrating that forcing overexpression of Nfatc1 in mouse primary chondrocytes reduced the expression of *Sox9* and *Col2α1* at day 3 and *Col10α1* (encoding the α1 chain of type X collagen) expression at day 21 [29]. Moreover, Ge et al. revealed that Nfatc1 restricts the proliferation and chondrogenesis of osteochondroma precursors [30]. Although there is no significant difference reported between *Nfatc1*-mutant and wild-type mice during normal cartilage development or in the post-traumatic osteoarthritis animal model, cartilage-specific ablation of *Nfatc1* in *Nfatc2^−/−^* mice markedly accelerated osteoarthritis development [31], which indicates that Nfatc1 may act as an osteoarthritis-suppressor. This study is the first report that clearly demonstrates the essential pro-chondrogenic role of Nfatc1 in mouse primary chondrocytes and reveals the potential underlying mechanism. In supporting the current understanding that “NFATs are good for your cartilage!” [32], our current discovery highlights the essential modulatory nature of Nfatc1 beyond its function as a regulator of inflammation [33] and enriches our knowledge about the myriad of complex interactions among a diverse group of growth factors and transcriptional factors during chondrogenesis.

## 4. Materials and Methods 

### 4.1. In Silico Promoter Analysis

Genomatix software suite v3.4 (Genomatix AG, Munich, Germany) was used to predict transcriptional factor binding motifs on the *Runx3* promoter. Sites were computationally projected with predefined transcription factor binding modules [34] in the Promoter Module Library (Genomatix).

### 4.2. Animal Maintenance

Mice were bred and maintained as previously described [5,15,20,35] under institutionally approved protocol provided by the Chancellor’s Animal Research Committee at UCLA (protocol number: 2012-041; 15 September 2012). *Runx2* haploinsufficient mice (*Runx2^+/−^* [35]) were mated with *Nell-1*-overexpressing mice (*CMV-Nell-1* [15]) to obtain *Runx2^−/−^/CMV-Nell-1* mice [20]. Due to the severe reduction of Nell-1 expression in homozygotes (*Nell-1^6R/6R^*; *Nell-1^6R^*: an ENU-induced point mutation truncating an 810 amino-acid Nell-1 protein at residue no. 502 [36,37]), neonatal death was induced [5]. Heterozygous *Nell-1^6R^* mice (*Nell-1^+/6R^*) were used to produce *Nell-1^6R/6R^* fetuses. Mouse genotypes were determined by PCR [5,15,20,35].

### 4.3. Immunofluorescence (IF) Staining

Animals used in this study were euthanized via phenobarbital (Piramal Healthcare, Maharashtra, India) overdose. Hind limbs, isolated from neonatal mice, were fixed in 4% paraformaldehyde at 4 °C overnight before paraffin embedding and sectioning at 5-μm. IF staining was performed following the instruction of ”abcam protocol book” (Abcam, available at http://docs.abcam.com/pdf/misc/abcam-protocols-book-2010.pdf). All primary antibodies were purchased from Abcam (Cambridge, MA, USA), IHC-Tek^TM^ Antibody Diluent pH7.4 (IHCWORD, Woodstock, MD, USA) and were used for blocking and antibody dilution; Dnk pAb to Rb IgG (Alexa Fluor^®^ 488) (ab150073, Abcam) was used as the second antibody and 2-(4-amidinophenyl)-1H-indole-6-carboxamidine (DAPI; Sigma-Aldrich, St. Louis, MO, USA) was used for nuclear counterstaining.

### 4.4. Cultivation of ATDC5 Cell Line

A well-known chondrogenic-committed cell line, ATDC5 [38], was firstly used for the candidate transcriptional factor screening. ATDC5 cells were obtained from the RIKEN Cell Bank (Tsukuba, Japan) and cultured in ATDC maintenance medium composed of a 1:1 mixture of Dulbecco’s modified Eagle’s medium (DMEM) and Ham’s F-12 medium containing 5% fetal bovine serum (FBS), 10 mg/mL of human transferrin (Sigma-Aldrich, St. Louis, MO, USA), and 30 nM sodium selenite (Sigma-Aldrich) at 37 °C in a humidified atmosphere consisting of 5% CO_2_ and 95% air. All cell culture media were purchased from Invitrogen (Carlsbad, CA, USA). Subconfluent ATDC5 cells were subjected to serum starvation (0.1% FBS) for 18 hours and stimulated with recombinant human Nell-1 protein for 3 h as previously described [8].

### 4.5. Mouse Primary Chondrocyte Isolation and Cultivation

Mouse primary chondrocyte isolation was conducted following the protocol provided by Dr. Karen Lyons’ lab at UCLA, which is available at: https://www.mcdb.ucla.edu/Research/Lyons/Protocols_files/Isolation_of_Sternal_Chondrocytes.pdf. Briefly, after removing soft tissues with 2 mg/mL protease (Roche, Nutley, NJ, USA) in PBS and 3 mg/mL collagenase II (Roche) in DMEM, rib cages of neonatal mouse embryos were incubated in 1 mg/mL collagenase II for 3 h to achieve single-cell suspension. After rinsing with DMEM, chondrocytes were cultured in a basal culture medium (DMEM with 10% FBS, 100 U/mL penicillin, and 100 μg/mL streptomycin). The medium was changed every 3 days and cells were passaged at 70–90% confluence [6,8].

5 × 10^4^ cells/well P2 chondrocytes were seeded in 6-well plates with basal culture medium for 6 h. Recombinant human Nell-1 protein was synthesized by Aragen Bioscience Inc. (Morgan Hill, CA, USA) with a purity of 92%. Before treatment, cells were synchronized by being cultured in a starvation medium (DMEM + 1% ITS Universal Cell Culture Supplement Premix (BD Biosciences, San Jose, CA, USA)) for 18 h [6,8].

For 3D micromass culture, cells were reconstituted in the culture medium at a density of 1 × 10^7^ cells/mL, and 10 μL of cell suspension was dropped into each well of a 24-well plate. The cell culture plates were incubated in a 37 °C incubator for 3 h before adding culture medium to allow cells to attach to the dish [6,8].

### 4.6. RNAi

Plasmid packages harboring shRNA targeting mouse *Nfatc1* and *Runx1*, respectively, were obtained from Origene (Rockville, MD, USA). For each package, there were four shRNA plasmids harboring different sequences against the target genes. P2 chondrocytes isolated from *Runx2^−/−^* mice were transfected with the shRNA plasmid with Lipofectamine 3000 reagent (Invitrogen). A control shRNA plasmid provided by OriGene was also used to transfect *Runx2^−/−^* mouse chondrocytes. Transfection efficiency was determined by qPCR.

### 4.7. ChIP Assay

ChIP assay was performed by using the Pierce Magnetic ChIP Kit (Thermo Fisher Scientific Inc., Waltham, MA, USA) following the manufacturer’s instruction. 4 × 10^6^ *Runx2^−/−^* chondrocytes were used per ChIP reaction. After being synchronized in the starvation medium for 18 h, cells were treated with Nell-1 for 12 h. 5 μg of a ChIP grade Nfatc1 primary antibody (Thermo Fisher Scientific Inc.) was used for each target-specific IP. qPCR analysis was performed to qualify the DNA fragments pulled down by antibodies. The primer sequences used are: −833–−810 locus: Forward: 5′-CTGGGG AAG AGT CAG CAG GTC A-3′, Reverse: 5′-GTG GAT CTG GCT TTA CCG TGG A-3′; −280–−257 locus: Forward: 5′-TGT GGG ACT GGG AAG CCA AAT-3′, Reverse: 5′-GGG AAA GAA CGC GAG AGC GT-3′.

### 4.8. qPCR

Total RNA was isolated by TRIzol^®^ Reagent (Invitrogen) followed by DNase (Invitrogen) treatment. 1 μg RNA was injected for reverse transcription (RT) with the SuperScript II Reverse Transcriptase Kit (Invitrogen). qPCR was performed on the 7300 Real-Time PCR system with SYBR Green Mastermix (Invitrogen). All the primer sequences used are listed in Table 2. Concomitant *glyceraldehyde 3-phosphate dehydrogenase* (*Gapdh*) was also evaluated in separate tubes for each RT reaction as a housekeeping standard. Relative gene expression was analyzed by _ΔΔ_C_T_ method [39].

### 4.9. Alcian Blue Staining and Quantification

Alcian Blue staining was performed by fixing micromass cultures at day 3 and then incubating them with 0.1% Alcian Blue, pH 2.5, for 2 h. Quantification of the staining was achieved after extensive washing with water by extraction with 6 M guanidine-hydrogen chloride for 8 h at room temperature. All chemicals were purchased from Sigma-Aldrich (St. Louis, MO, USA). Dye concentrations were determined spectrophotometrically at A_630_ [40].

### 4.10. Statistical Analysis

All statistical analyses were conducted in consultation with the UCLA Statistical Biomathematical Consulting Clinic. ANOVA and two-sample *t*-tests were computed by OriginPro 8 (Origin Lab Corp., Northampton, MA, USA) for statistical analysis. *p*-Value < 0.05 was considered statistically significant.

## 5. Conclusions

In this study, we identified and functionally validated Nfatc1 as a key transcriptional factor mediating Nell-1 → Runx3 signal transduction in chondrocytes by binding to the -833 - -810 region of *Runx3*-promoter. It is the first report that clearly demonstrates the essential pro-chondrogenic role of Nfatc1 in mouse primary chondrocytes and reveals the potential underlying mechanism. As an additional section of a series of investigations that uncover the underlying mechanism of Nell-1’s function in chondrogenesis, this study provides innovative insights into developing a novel therapeutic platform for managing cartilage regeneration and other chondrogenesis-related conditions.

## Figures and Tables

**Table 1:** Mouse Runx3 promoter binding transcriptional factor candidates that expressed in cartilage and chondrocytes.

In Silico Bioinformatics Predicted Mouse *Runx3* Promoter Binding Transcriptional Factors That Expressed in Cartilage and Chondrocytes						Gene Expression Changes in ATDC5 Cells Due to the Nell-1 Treatment *	
Matrix Family	Gene	Full Name	Strand	Sequence **	Matrix Similarity	Fold-Change	*p*-Value
FKHD	***Foxp1***	Forkhead box P1	(−)	5′-gggtcaaAACAgagggg-3′	1	1.14	5.7 × 10^−6^
	***Foxp2***	Forkhead box P2	(+)	5′-cagcagtaAACAgagag-3′	0.994	0.79	1.1 × 10^−2^
	***Hnf3b***/***Foxa2***	Hepatic nuclear factor 3 beta	(+)	5′-cagcagtaAACAgagag-3′	0.914	0.32	2.0 × 10^−2^
	***Foxo1***	Forkhead box protein O1	(+)	5′-aaaaagtcAACAcctcc-3′	0.9	0.73	7.9 × 10^−5^
	***Fhx***/***Foxj2***	Fork head homologous X binds DNA with a dual sequence specificity (FHXA and FHXB)	(+)	5′-gtagccACAAgatcttc-3′	0.835	0.84	2.6 × 10^−5^
HAML	***Runx1***	Runt-related transcription factor 1	(+)	5′-ctgtGTGGtccggac-3′	0.97	1.23	8.8 × 10^−5^
HOMF	***Hhex***	Hematopoietically expressed homeobox, proline-rich homeodomain protein	(+)	5′-aactaggtgttTAATtttg-3′	0.969	0.90	3.0 × 10^−4^
			(+)	5′-ttcccaccattTAATgata-3′	0.952		
	***Hmx3***	H6 homeodomain HMX3/Nkx5.1 transcription factor	(+)	5′-cggaccccAAGTgcctcca-3′	0.897	2.99	0.19 ***
			(+)	5′-ggctcaggAAGTgggggtg-3′	0.911		
			(+)	5′-agccaaccAAGTgggtctg-3′	0.96		
	***Msx-1***	Homeodomain proteins MSX-1 and MSX-2	(+)	5′-aggtgttTAATtttgcaac-3′	0.989	0.81	4.5 × 10^−4^
	***Hmx2***	Hmx2/Nkx5-2 homeodomain transcription factor	(−)	5′-gtatcaTTAAatggtggga-3′	0.86	0.90	0.69 ***
	***Tlx1***/***Hox11***	T-cell leukemia, homeobox 1	(−)	5′-tgggagcCGCTgagtgggt-3′	0.858	1.41	3.0 × 10^−2^
NFAT	***Nfatc1***	Nuclear factor of activated T-cells, cytoplasmic, calcineurin-dependent 1, dimeric binding site	(−)	5′-tttaccGTGGaacccagga-3′	0.826	1.55	5.3 × 10^−4^
			(+)	5′-ggttccACGGtaaagccag-3′	0.816		
			(−)	5′-atctccAAGGaaagaaagt-3′	0.827		
			(+)	5′-ctttccTTGGagattttct-3′	0.877		
	***Nfat5***	nuclear factor of activated T-cells 5	(−)	5′-ccaaGGAAagaaagtttcg-3′	0.844	1.52	6.0 × 10^−5^
SORY	***Sox1***	SRY (sex determining region Y)-box 1, dimeric binding sites	(−)	5′-gctGATTccccactcaggcagag-3′	0.795	0.43	0.14 ***
	***Sox21***	SRY (sex determining region Y)-box 21, dimeric binding sites	(−)	5′-cctGCATgttggtcacacaacta-3′	0.783	ND ****	ND ****
			(+)	5′-attTAATgatactctgcacatag-3′	0.762		
	***Sox2***	SRY-box containing gene 2, dimeric binding sites	(−)	5′-gatCAAGggtgtgaatagagtcc	0.701	0.90	8.7 × 10^−6^
	***Sox8***	SRY (sex determining region Y)-box 8, dimeric binding sites	(−)	5′-aatGAAAggcagtactgacctgc-3′	0.777	ND ****	ND ****
			(+)	5′-cagGACTcccagtctcacagggt-3′	0.763		
	***Hbp1***	high mobility group box transcription factor 1	(−)	5′-aatgaatgAATGaacgaggctca-3′	1	0.84	3.8 × 10^−5^
			(−)	5′-gatgaatgAATGaatgaacgagg-3′	1		
			(−)	5′-ctggatgAATGaatgaatgaacg-3′	0.996		
			(−)	5′-cagcctgGATGaatgaatgaatg-3′	0.847		
	***Sox9***	SRY (sex determining region Y)-box 9 homodimer	(+)	5′-acAGAAagcctaccttctctctc-3′	0.787	0.76	1.1 × 10^−5^
			(+)	5′-gaaccACAAggccaggccctcgc-3′	0.944		
			(−)	5′-gcaCTATgtgcagagtatcatta-3′	0.733		
	***Hmgiy***/***Hmga1***	HMGI(Y) high-mobility-group protein I (Y), architectural transcription factor organizing the framework of a nuclear protein-DNA transcriptional complex /High mobility group AT-Hook 1	(−)	5′-cacaAATTttcaacagcactatg-3′	0.935	0.56	1.5 × 10^−3^
			(+)	5′-tgaaAATTtgtggctagacattc-3′	0.935		
	***Sox10***	SRY-box containing gene 10	(−)	5′-caGGAAtgtctagccacaaattt-3′	0.739	8.25	0.54 ***
PAX	***Pax1***	Pax1 paired domain protein, expressed in the developing vertebral column of mouse embryos	(−)	5′-cTGTTttgttatatatatt-3′	0.667	ND ****	ND ****

* Data are subtracted from the GEO DataSet: GSE23570; ** Capitalized characters represent the core sequence; *** Not statically significant; **** Not detected in the published dataset (GEO DataSet: GSE23570) using Affymetrix Mouse Genome Array.

**Table 2:** Primer sequences used for real-time PCR.

Gene	Primer Sequence
***Acan***	5′-CCA GGC TCC ACC AGA TAC TC-3′
	5′-TGC TCA TAG CCT GCC TCA TA-3′
***Col2α1***	5′-GTC CTG AAG GTG CTC AAG GT-3′
	5′-TTT GGC TCC AGG AAT ACC AT-3′
***Gapdh***	5′-ATT CAA CGG CAC AGT CAA GG-3′
	5′-GAT GTT AGT GGG GTC TCG CTC-3′
***Gli1***	5′-CCA AGC CAA CTT TAT GTC AGG G-3′
	5′-AGC CCG CTT CTT TGT TAA TTT GA-3′
***Ihh***	5′-CTC AGA CCG TGA CCG AAA TAA G-3′
	5′-CCT TGG ACT CGT AAT ACA CCC AG-3′
***Nfat5***	5′-TGC TTT CTC AGC TTA CCA CGG-3′
	5′-GTC CGC ACA ACA TAG GGC TC-3′
***Nfatc1***	5′-GGA GAG TCC GAG AAT CGA GAT-3′
	5′-TTG CAG CTA GGA AGT ACG TCT-3′
***Ptch 1***	5′-TGC CAC AGC CCC TAA CAA AAA-3′
	5′-ACC CAC AAT CAA CTC CTC CTG-3′
***Runx1***	5′-ACG ATG AAA ACT ACT CGG CAG-3′
	5′-CTG AGG TCG TTG AAT CTC GCT-3′
***Runx3***	5′-CAG GTT CAA CGA CCT TCG ATT-3′
	5′-GTG GTA GGT AGC CAC TTG GG-3′
***Sox9***	5′-ACG GCT CCA GCA AGA ACA AG-3′
	5′-TTG TGC AGA TGC GGG TAC TG-3′
***Tlx1***	5′-CGG CTT GCC TAC AGT ACC C-3′
	5′-CTG CGG TTA CTC TCC ATC CAG-3′

## Abbreviations

Acan	Aggrecan
ChIP	Chromatin immunoprecipitation
DAPI	2-(4-amidinophenyl)-1H-indole-6-carboxamidine
DMEM	Dulbecco’s modified Eagle’s medium
ENU	*N*-ethyl-*N*-nitrosourea
FBS	Fetal bovine serum
Gapdh	Glyceraldehyde 3-phosphate dehydrogenase
IF	Immunofluorescent
Ihh	Indian hedgehog
Nfat5	Nuclear factor of activated T-cells 5
Nfatc1	Nuclear factor of activated T-cells 1
Patched1	Patched homology 1
qPCR	Quantitative real-time PCR
RNAi	RNA interference
RT	Reverse transcription
Runx1	Runt-related transcription factor 1
Runx2	Runt-related transcription factor 2
Runx3	Runt-related transcription factor 3
Tlx1	T-cell leukemia, homeobox1
